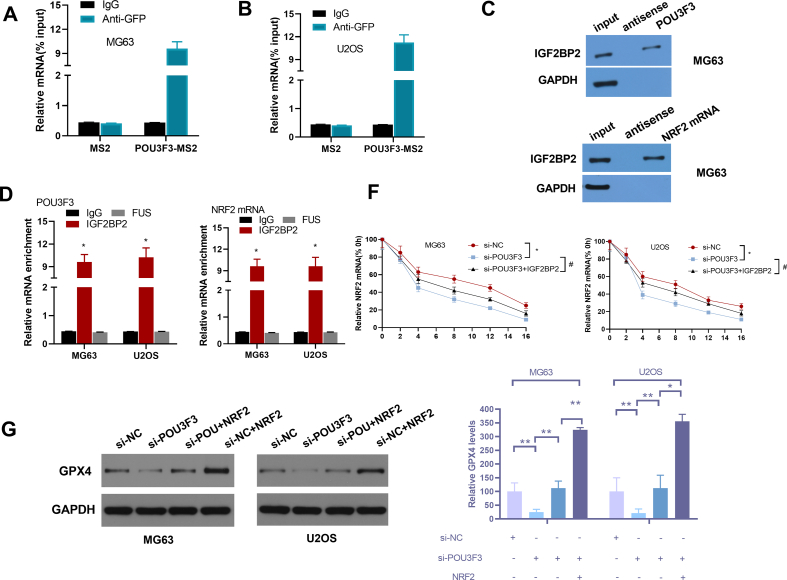# lncRNA POU3F3 promotes osteosarcoma progression through GPX4-modulated ferroptosis by interaction with IGF2BP2 to facilitate NRF2 mRNA stability

**DOI:** 10.1016/j.gendis.2024.101439

**Published:** 2024-10-12

**Authors:** Suyang Xu, Yingjie Zhou, Ma Wan, Yikang Bi, Pengcheng Liu, Zixiang Li, Yafeng Xu, Hao Fang, Hai Hu, Chongshan Yang, Shenghui Lan

**Affiliations:** a Department of Orthopaedics, The Eighth People's Hospital, Jiangsu University, Shanghai 200235, China; b Department of Orthopaedics, Xuhui Branch of The Sixth People's Hospital, Shanghai Jiao Tong University, Shanghai 200233, China; c Department of Anesthesiology, Dongfang Hospital of Beijing University of Chinese Medicine, Beijing 100078, China; d Department of Orthopaedics, Suzhou Wujiang District Hospital of Traditional Chinese Medicine (Suzhou Wujiang District Second People's Hospital), Suzhou 215000, China

Osteosarcoma (OS) is a highly aggressive bone tumor that predominantly affects teenagers and young adults, characterized by rapid growth and a high propensity for metastasis.^1^ Despite significant advancements in surgical techniques and chemotherapy, the survival rate for OS patients has plateaued over the past decades.^2^ Understanding the molecular mechanisms underlying OS progression and identifying new therapeutic targets remain critical for improving patient outcomes. Long non-coding RNAs (lncRNAs) are a class of functional RNA molecules that are typically over 200 nucleotides in length and cannot code for proteins.^3^ Initially considered as “junk DNA”, recent studies have revealed the crucial roles lncRNAs play in a wide range of biological processes, including cellular differentiation, apoptosis, inflammation, and cancer.^4^

The additional results and detailed materials & methods were shown as supplementary data. This study aimed to investigate the role of lncRNA POU3F3 (POU3F3) in OS, particularly its impact on cell growth, invasion, migration, and sensitivity to ferroptosis, an iron-dependent form of programmed cell death. Our research showed that POU3F3 was significantly up-regulated in OS cell lines versus normal osteoblasts. Knocking down POU3F3 in high-expressing OS cell lines (MG63 and U2OS) suppressed cell proliferation, increased apoptosis, and reduced invasion and migration capacities (Fig. S1, 2). These findings suggest that POU3F3 plays a crucial role in OS cell survival and metastatic potential.

Notoriously, ferroptosis contributes to cancer development and progression, although its molecular mechanism in epigenetics is not yet fully understood.^5^ To further elucidate the role of POU3F3 in ferroptosis, we treated POU3F3-inhibited OS cells with ferroptosis inducers, Erastin and RSL3. POU3F3 knockdown cells exhibited heightened susceptibility to ferroptosis, indicated by increased intracellular iron and malondialdehyde levels and decreased glutathione levels and glutathione peroxidase (GPX4) activities (Fig. S3). Mechanistically, POU3F3 interacts with insulin-like growth factor 2 mRNA-binding protein 2 (IGF2BP2), stabilizing nuclear factor erythroid 2-related factor 2 (NRF2) mRNA and enhancing the expression of the ferroptosis inhibitor GPX4 (Fig. S4, 5). Rescue experiments confirmed that GPX4 overexpression counteracted the effects of POU3F3 knockdown on cell proliferation, invasion, and ferroptosis, highlighting the POU3F3/IGF2BP2/NRF2/GPX4 axis as a critical pathway in OS pathogenesis.

The data supporting these conclusions were obtained through various assays. Quantitative real-time PCR was used to measure the expression levels of POU3F3 in OS and normal cell lines. Cell proliferation was assessed using CCK-8 and EdU assays, and apoptosis was evaluated via flow cytometry. OS cells' invasion and migration capacities were determined using transwell and wound healing assays. Ferroptosis sensitivity was examined by treating cells with Erastin or RSL3 and measuring cell viability, intracellular iron, malondialdehyde, glutathione levels, and GPX4 activity.

Further mechanistic studies revealed that POU3F3 interacted with IGF2BP2, which bound to NRF2 mRNA and enhanced its stability. This interaction led to increased expression of NRF2 and its downstream target GPX4, a key regulator of ferroptosis. By stabilizing NRF2 mRNA, POU3F3 up-regulated GPX4 expression, thereby protecting OS cells from ferroptosis. Rescue experiments demonstrated that overexpressing GPX4 in POU3F3-knockdown cells could reverse the observed effects on cell proliferation, invasion, and ferroptosis sensitivity, confirming the involvement of the POU3F3/IGF2BP2/NRF2/GPX4 axis in OS pathogenesis (Fig. 1).

**Figure 1:**
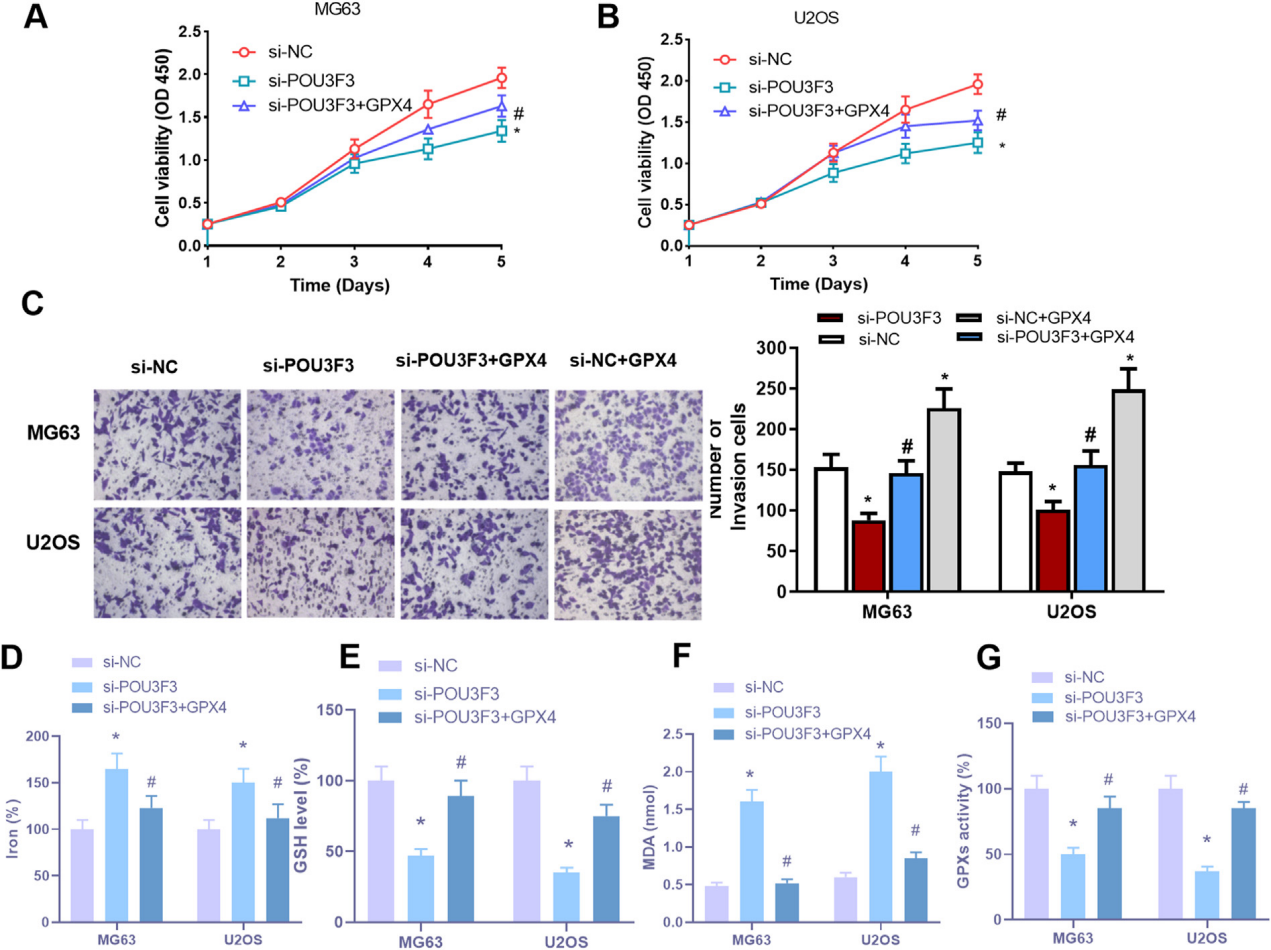
POU3F3 facilitated proliferation and inhibited ferroptosis of OS cells in a GPX4-dependent manner. **(A, B)** CCK8 assays demonstrated the rescue effect of GPX4 (glutathione peroxidase 4) overexpression on cell proliferation reduction caused by POU3F3 knockdown. **(C)** Transwell assays demonstrated the rescue effect of GPX4 overexpression on cell invasion reduction caused by POU3F3 knockdown. **(D)** Impact of GPX4 overexpression on elevated iron levels observed after POU3F3 inhibition. **(E)** Reversal of glutathione (GSH) depletion upon GPX4 overexpression following POU3F3 knockdown. **(F)** Suppression of elevated malondialdehyde (MDA) production upon GPX4 overexpression following POU3F3 inhibition. **(G)** Recovery of baseline glutathione peroxidase (GPX) activity upon GPX4 overexpression following POU3F3 inhibition.

The implications of these findings are significant, as they not only provide a deeper understanding of the molecular mechanisms underlying OS but also identify potential biomarkers and therapeutic targets. Given the complexity of OS and the poor prognosis associated with metastatic disease, targeting the POU3F3/IGF2BP2/NRF2/GPX4 axis could offer new avenues for treatment, potentially improving survival rates and quality of life for OS patients.

In conclusion, our study identifies POU3F3 as a critical regulator of OS cell behavior and ferroptosis sensitivity. By modulating the POU3F3/IGF2BP2/NRF2/GPX4 axis, POU3F3 promotes oncogenic activities in OS cells, providing a promising target for future therapeutic strategies. Further research is warranted to explore the clinical applications of these findings and to develop targeted therapies that can effectively inhibit POU3F3 function in OS.

## Conflict of interests

The authors declared no conflict of interests.

## Funding

This work was supported by grants from the 10.13039/501100001809National Natural Science Foundation of China (No. 81601902), the 10.13039/501100002858China Postdoctoral Science Foundation (No. 2015M572817), the 10.13039/501100003819Natural Science Foundation of Hubei Province, China (No. 2015CFB240), and the Research Fund of Shanghai Municipal Health Commission for Clinical Research in Medical Science (China) (No. 202040084).

## Figures and Tables

**figs1:**
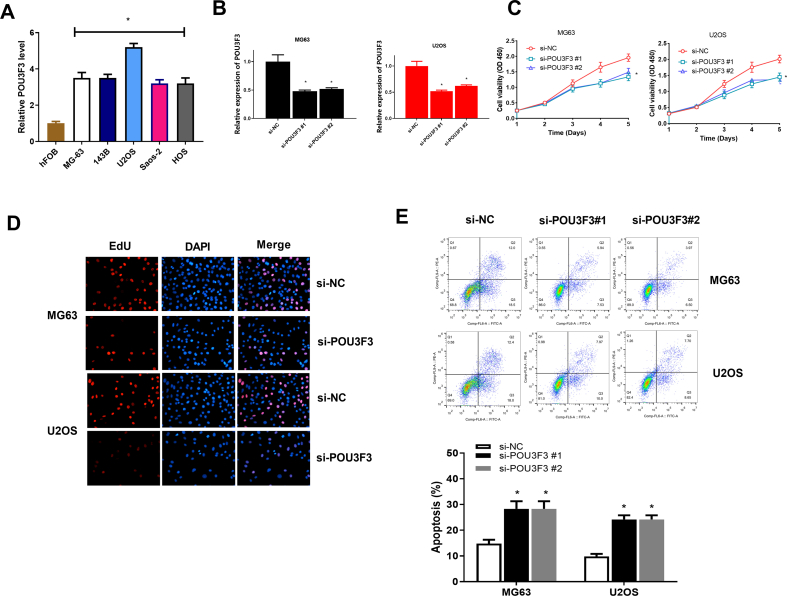


**figs2:**
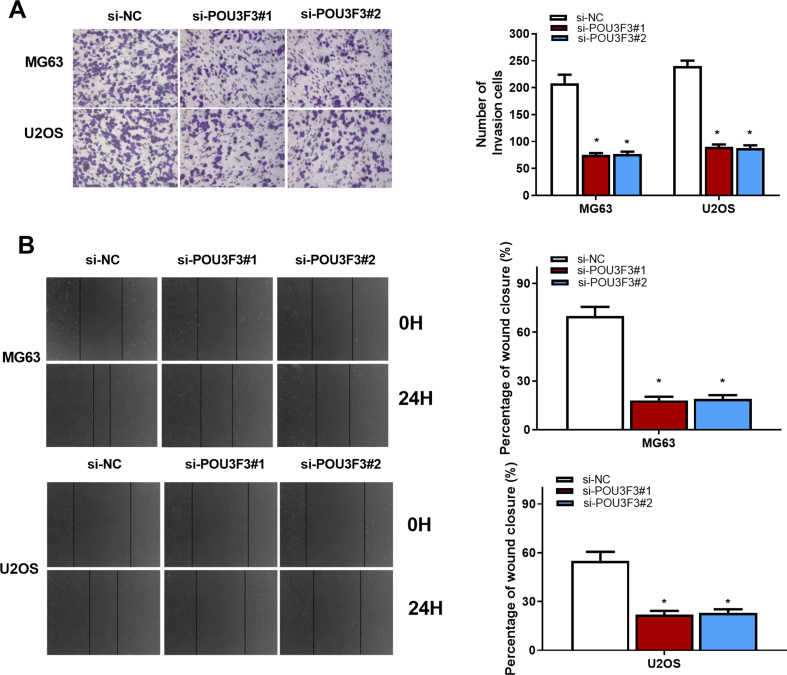


**figs3:**
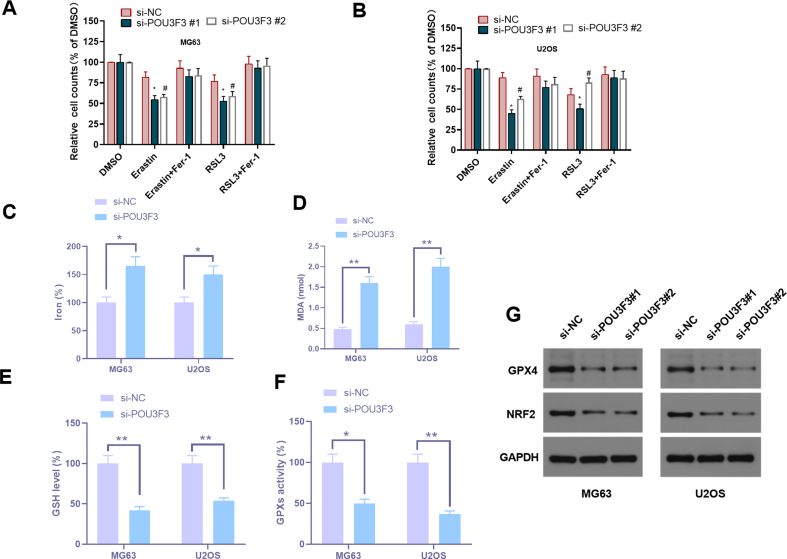


**figs4:**
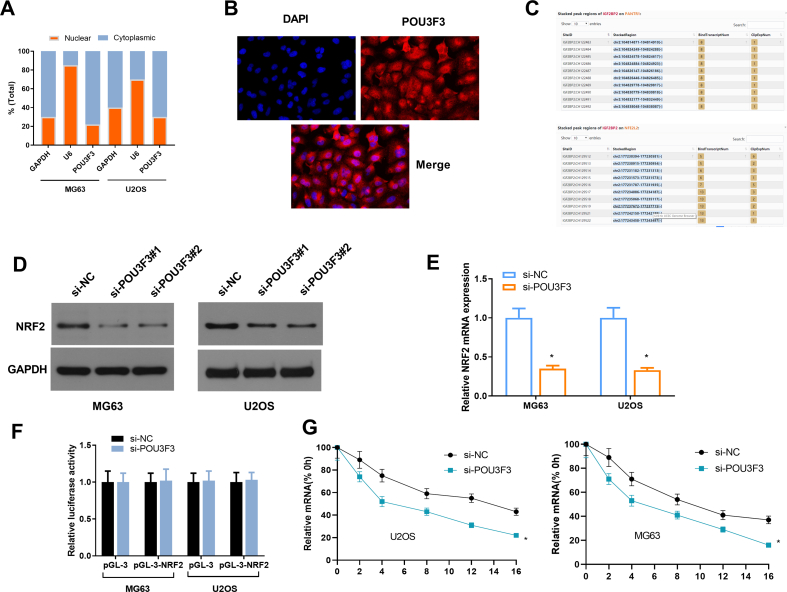


**figs5:**