# Supplementary material for: lncRNA POU3F3 promotes osteosarcoma progression through GPX4-modulated ferroptosis by interaction with IGF2BP2 to facilitate NRF2 mRNA stability
Source: Genes Dis. 2024 Oct 12;12(5):101439. doi: 10.1016/j.gendis.2024.101439 (PMC12099908; doi:10.1016/j.gendis.2024.101439)
Supplement: Multimedia component 1 [file mmc1.docx]

**Materials and methods**

**Cell culture**

The Shanghai Institutes for Biological Sciences provided the human osteoblasts cell line (hFOB) and a number of human osteosarcoma cell lines (MG63, U2OS, Saos-2, HOS, 143B). The F12K medium from WISENT (Canada) was used to cultivate MG63 cells, while Dulbecco's modified Eagle's medium (DMEM H-21 4.5 g/Liter glucose, WISENT, Canada) was used to cultivate U2OS cells. The remaining cell lines, however, were grown in WISENT (Canada) RPMI 1640 media. 10% bovine serum from WISENT (Canada) and 1% penicillin/streptomycin from Gibco (CA, USA) were added to all culture medium as supplements. Every cell line was cultured at 37 °C with 5% CO2 in a humidified cell incubator.

**Quantitative real-time polymerase chain reaction (qRT-PCR)**

TRIzol reagent from Invitrogen (Carlsbad, CA, USA) was used as directed to purify the total RNA from tissues or cells. The nuclear and cytoplasmic fractions were separated using Thermo Fisher Scientific's (MA, USA) NE-PER Nuclear and Cytoplasmic Extraction Reagents. Thermo Fisher Scientific's (MA, USA) NanoDrop ND-2000 spectrophotometer was used to measure the concentration and purity of the isolated RNA. Using Proteinbio's TRUE script RT Kit, 1 μg of pure RNA was reverse transcribed for cDNA synthesis (Nanjing, China). The 2× Universal SYBR Green qPCR Supermix from Proteinbio (Nanjing, China) was then used for qRT-PCR using an Applied Biosystems 7500 Real-Time PCR System (Waltham, MA, USA). For lncRNAs and mRNAs located in the cytoplasm and nucleus, respectively, the expression levels of GAPDH and small nuclear U6 were used as internal benchmarks. The sequences of all primers, which were acquired from RiboBio (Guangzhou, China), are listed in Table S1 (Supplementary Table S1).

**Cell Transfection**

In order to produce stable POU3F3-knockdown OS cell lines, GeneChem (Shanghai, China) provided a human lentivirus called sh-POU3F3-AS1. Table S2 contains the relevant sequences for the specific small interference RNAs (siRNAs) that RiboBio (Guangzhou, China) chemically synthesised and targeted against POU3F3. The Lipofectamine 3000 manufacturer's protocol was adhered to throughout the transfection process. Stably transfected cell lines were selected using puromycin, a selection agent that eliminates non-transfected cells and allows the survival and expansion of the transfected cells.

**Cell proliferation assay**

Utilising a CCK-8 test kit (Dojindo Laboratories Co. Ltd., Kumamoto, Japan), the cell proliferation assay was carried out. In a nutshell, 100μL of RPMI-1640 culture media per well was used to seed cells (2×10^3^ cells/well) in 96-well plates. 10% FBS was used as a supplement, and the cells were then cultivated at 37 °C in 5% CO2 environment. There are six duplicates of each sample. 100μL new culture media was used in lieu of the old medium, and 10μL of CCK-8 solution was added to each well for varying lengths of time (6, 24, 48, 72, and 96 hours). Using a Quant ELISA Reader (BioTek Instruments, USA), absorbance was measured spectrophotometrically at 450 nm after a 2-hour incubation period. Every experiment was carried out once and in quintuplicate.

**Western blot**

Using RIPA lysis buffer, the total protein from OS tissues and cells was purified. Leagene Biotechnology's (Beijing, China) BCA protein assay kit was used to measure the protein's concentration. Lysates of OS cells or tissue samples were then separated using SDS-PAGE and put onto PVDF (polyvinylidene difluoride) membranes. The membranes were exposed to 5% evaporated milk for one hour at room temperature in order to block them. Following that, primary antibodies were added to the membranes and incubated for around 12 hours at 4 °C. After that, the membranes underwent three rounds of TBST buffer washing before being incubated for two hours at room temperature with secondary antibodies conjugated with HRP. Ultimately, a BioSpectrum 600 Imaging System from Thermo Fisher (MA, USA) helped to visualise the blots on the membranes using ECL chemiluminescent reagents from Millipore (MA, USA).

**Transwell assay and wound healing assay**

Corning (NY, USA) chambers were used to test the OS cells' ability to migrate and invade. In some chambers, Matrigel from BD Biosciences (New Jersey, USA) was used as an additive, whereas in other chambers, Matrigel was kept out. The chambers' dimensions allowed them to be put on a 24-well plastic plate. 800 μL of media containing 10% FBS was introduced to the bottom chamber. Next, 200 μL of serum-free media containing MG-63 and U2OS cells (3×10^4^) suspended were added to the top chamber. For 48 hours, the cells were left to incubate. Following the incubation time, OS cells that stuck to the membrane were fixed with paraformaldehyde and stained with 0.1% crystal violet. The culture media was discarded. Any last cells were carefully cleaned out of the top chamber using cotton. Under a microscope, the OS cells that had attached themselves to the membrane in the bottom compartment were examined. Six-well plates containing treated cells were used for the wound healing experiments. A sterile pipette tip was used to create the wound, and pictures were taken under a microscope at random times between 0 and 24 hours later.

**5-Ethynyl-2’-deoxyuridine (EdU) assay**

The supplier of the EdU reagent was RiboBio, located in Guangzhou, China. Following a 24-hour fixation period and washing, the transfected cells were grown on confocal dishes. After that, the cells were incubated with the EdU dye agent for 25 minutes, treated for 10 minutes with 0.2% Triton X-100 (Boster, Wuhan, China), and stained for 10 minutes with DAPI. At last, pictures were taken using a fluorescence microscope.

**Measurement of cellular ferroptosis levels**

Following the guidelines provided by the manufacturer, the glutathione (GSH) concentrations were measured using a glutathione assay kit (Solarbio, Beijing, China). Following the manufacturer's instructions, the amounts of malondialdehyde (MDA) were measured using a lipid peroxidation test kit (Solarbio, Beijing, China). Using an iron assay kit (Solarbio, Beijing, China) and following the manufacturer's instructions, iron concentrations were measured. Using a GPXs Assay Kit (Solarbio, Beijing, China) in accordance with the manufacturer's instructions, relative GPX activity was measured.

**Luciferase reporter assays**

In 24-well plates, MG63 and U2OS cells were cultivated. Subsequently, si-POU3F3 and matching NC mimics were co-transfected into several cell lineages. The cells were lysed after 48 hours, and the lysate contained firefly and Renilla luciferase activity. Relative activity was measured using firefly luciferase activity as a control.

**RNA pulldown assay**

Using T7 RNA polymerase from Promega (Madison, Wisconsin, USA), expression vectors containing the full-length POU3F3 sequence and its truncated fragments were created and transcribed in vitro. POU3F3 and the shortened fragments were biotinylated using Roche's Biotin RNA Labelling Mix kit (Basel, Switzerland, USA) after in vitro transcription. The Pierce Magnetic RNA-Protein Pull-Down Kit from Thermo Fisher Scientific (Waltham, MA, USA) was used to perform a pulldown test. For one hour, streptavidin magnetic beads were treated with biotin-labeled RNA at room temperature. Protease/Phosphatase Inhibitor Cocktail and RNase inhibitors were added at each stage of the procedures to guarantee protein stability and stop RNA degradation, and whole cell lysates were made fresh. Cell lysates were then treated with the complexes created by the beads and probes for six hours at 4 °C. The SDS buffer was added and boiling was used to elute the RNA-protein binding complexes after a thorough washing process to remove unattached proteins. The eluted proteins were analyzed using silver staining, western blotting, and Mass Spectrometry analysis to detect and identify the proteins involved in the RNA-protein interactions.

**RNA immunoprecipitation (RIP) assay**

Using the supplied instruction booklet, the EZ-Magna RIP Kit from Millipore (Bedford, MA, USA) was used for the RIP assay. For every immunoprecipitation, 100 μL of RIP lysis solution was used to lyse around 2×10^7^ MG63 cells. Protein A/G Magnetic Beads were treated with 5 μg of the target protein-specific antibody or the equivalent IgG control antibody for 30 minutes at room temperature in order to accomplish the immunoprecipitation. The antibody was then allowed to bind to the target protein and associated RNA by mixing 100 μL of the RIP lysates with the beads-antibody combination in a RIP Immunoprecipitation Buffer and incubating for 12 hours at 4 °C. Lastly, quantitative real-time polymerase chain reaction (qRT-PCR) was used to identify and evaluate the extracted RNA in order to determine its amount and presence.

**RNA stability assay**

Actinomycin D (2 mg/ml) was used to treat MG63 and U2OS cells in order to evaluate the stability of NRF2 mRNA. Actinomycin D was purchased from Sigma-Aldrich (St. Louis, MO, USA). Total RNA was collected from MG63 and U2OS cells at predetermined intervals (0, 8, 16, 24 h) after the treatment. The relative amount of NRF2 mRNA that remained was determined using quantitative real-time polymerase chain reaction (qRT-PCR). The mRNA levels at each time point were compared to the initial mRNA level (0 h) to evaluate the degradation rate and stability of NRF2 mRNA over time.

**Supplementary table legends**

**Table S1** Primer sequence

| Target name | Forward primer (5′-3′) | Reverse primer (5′-3′) |
| --- | --- | --- |
| GAPDH | CTGCCCCCTCTGCTGATG | TCCACGATACCAAAGTTGTCATG |
| U6 | CTCGCTTCGGCAGCACATATACT | CGCTTCACGAATTTGCGTGT |
| NRF2 | TGCTTTATAGCGTGCAAACCTCGC | AATCCATGTCCCTTGACAGCACAGA |
| POU3F3 | TCATCCTTCAGRGRCCATCC | ATCTCAGATTCCTGGGCTGG |

**Table S2** The relevant sequences for the specific small interference RNAs (siRNAs) that chemically synthesized and targeted against POU3F3

| POU3F3 siRNA |
| --- |
| 1# 5′- CTCTTATCGATTGATTAGGCA -3′; |
| 2# 5′- CTATATGCATGGGTTATCATA -3′; |

**Supplementary figure legends**


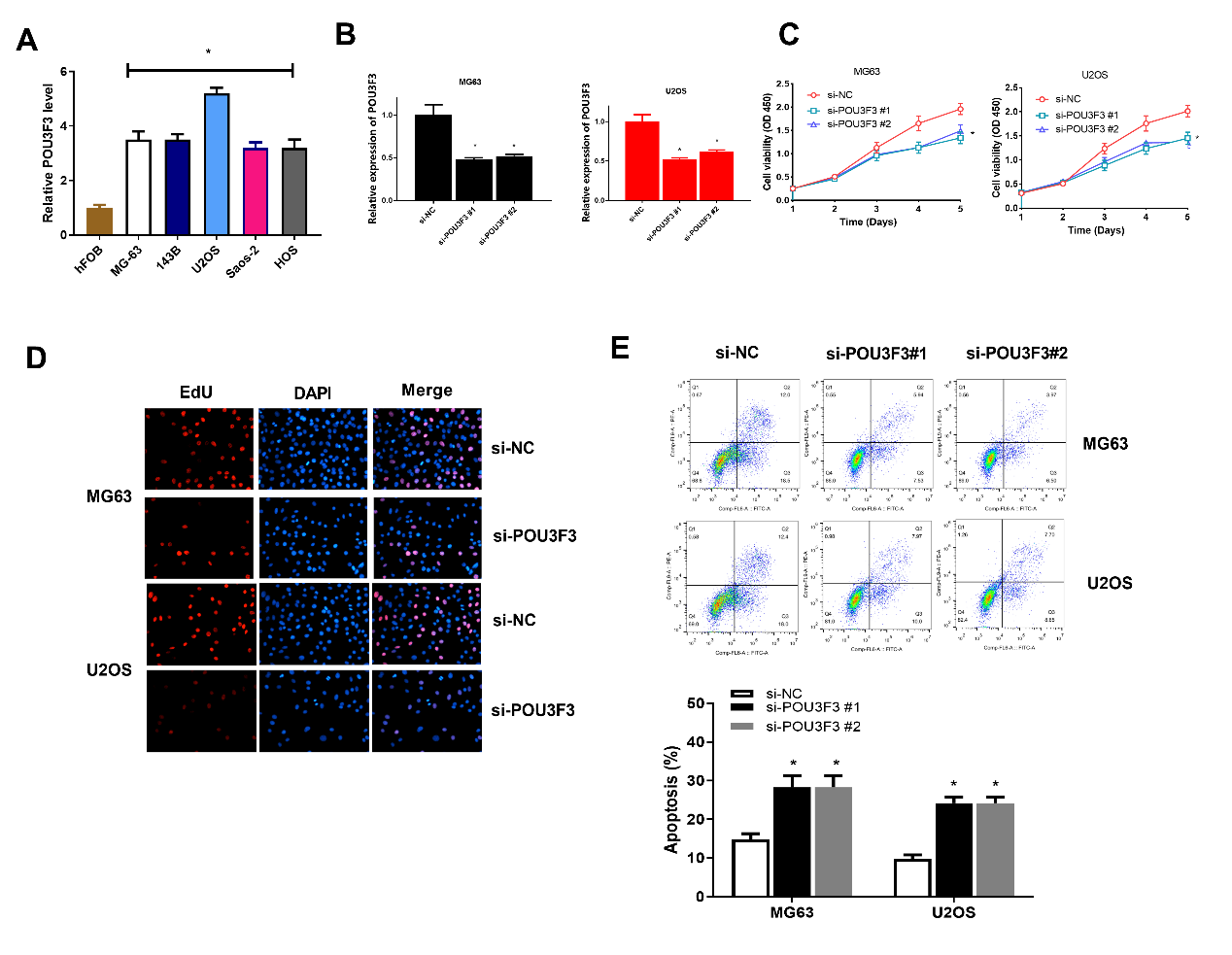


**Supplementary figure 1** POU3F3 promoted the growth and survivalof OS cells. (A) Representative expression analysis of POU3F3 in osteosarcoma cell lines, highlighting aberrantly high expression levels of POU3F3. (B) Evaluation of POU3F3 knockdown efficiency using qRT-PCR in MG63 and U2OS osteosarcoma cell lines. (C) Illustration of cell proliferation assays (CCK-8) performed to assess the effects of POU3F3 knockdown. (D) Illustration of cell proliferation assays (EdU) performed to assess the effects of POU3F3 knockdown. (E) Application of flow cytometry to quantify apoptotic changes induced by POU3F3 depletion in osteosarcoma cells.


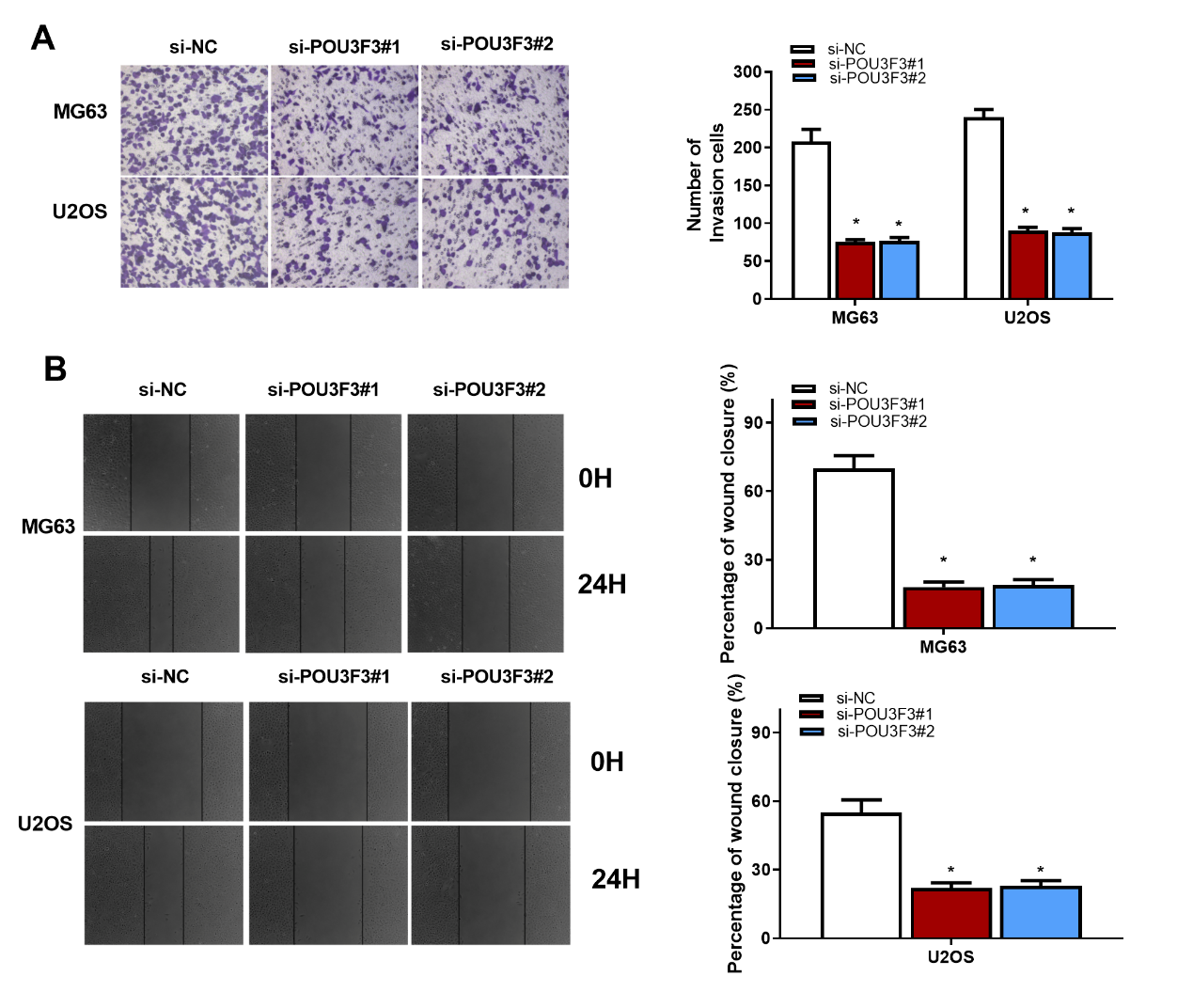


**Supplementary figure 2** POU3F3 promoted the invasion and migration of OS cells. (A) Examination of cell invasion capability following POU3F3 knockdown in osteosarcoma cells by transwell assay. (B) Examination of cell migration capability following POU3F3 knockdown in osteosarcoma cells by wound healing assay.


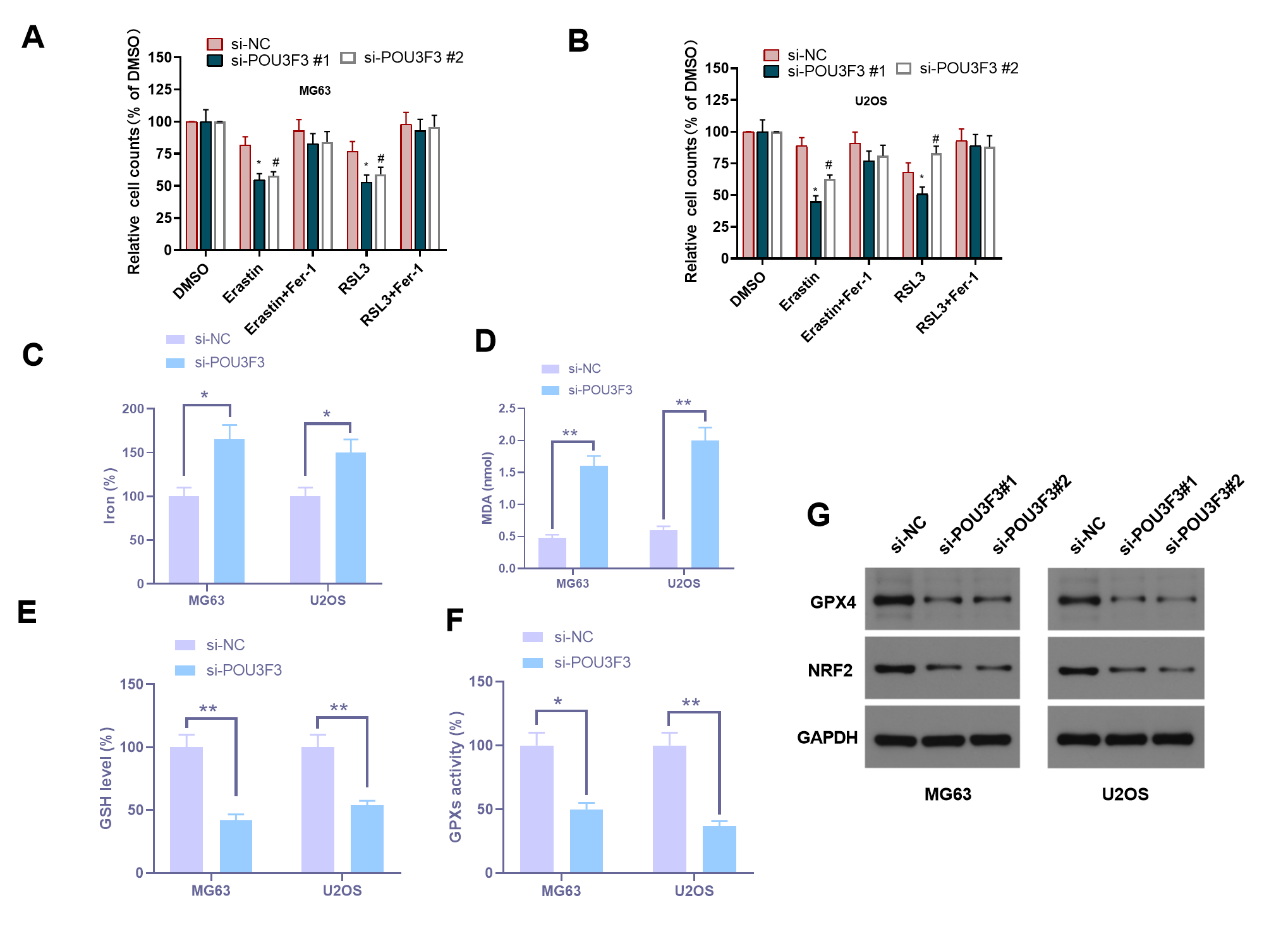


**Supplementary figure 3** POU3F3 suppressed ferroptosis and promoted NRF2\GPX4 expression in OS cells. (AB) Viability comparison of MG63 cells (A) and U2OS cells (B) treated with erastin or RSL3, with or without POU3F3 inhibition (si-POU3F3). (C)Quantification of intracellular Iron levels in MG63 cells with or without POU3F3 inhibition (si-POU3F3), indicating increased ferroptosis susceptibility. (D) Measurement of MDA production in OS cells following POU3F3 inhibition (si-POU3F3), demonstrating increased oxidative stress. (EF) Evaluation of cellular glutathione (GSH) levels (E) and GPX4 activity changes (F) after POU3F3 inhibition, indicating compromised GSH synthesis and GPX4 function. (G) Western blot analysis revealing reduced levels of GPX4, a ferroptosis marker, and NRF2 in cells with POU3F3 inhibition.


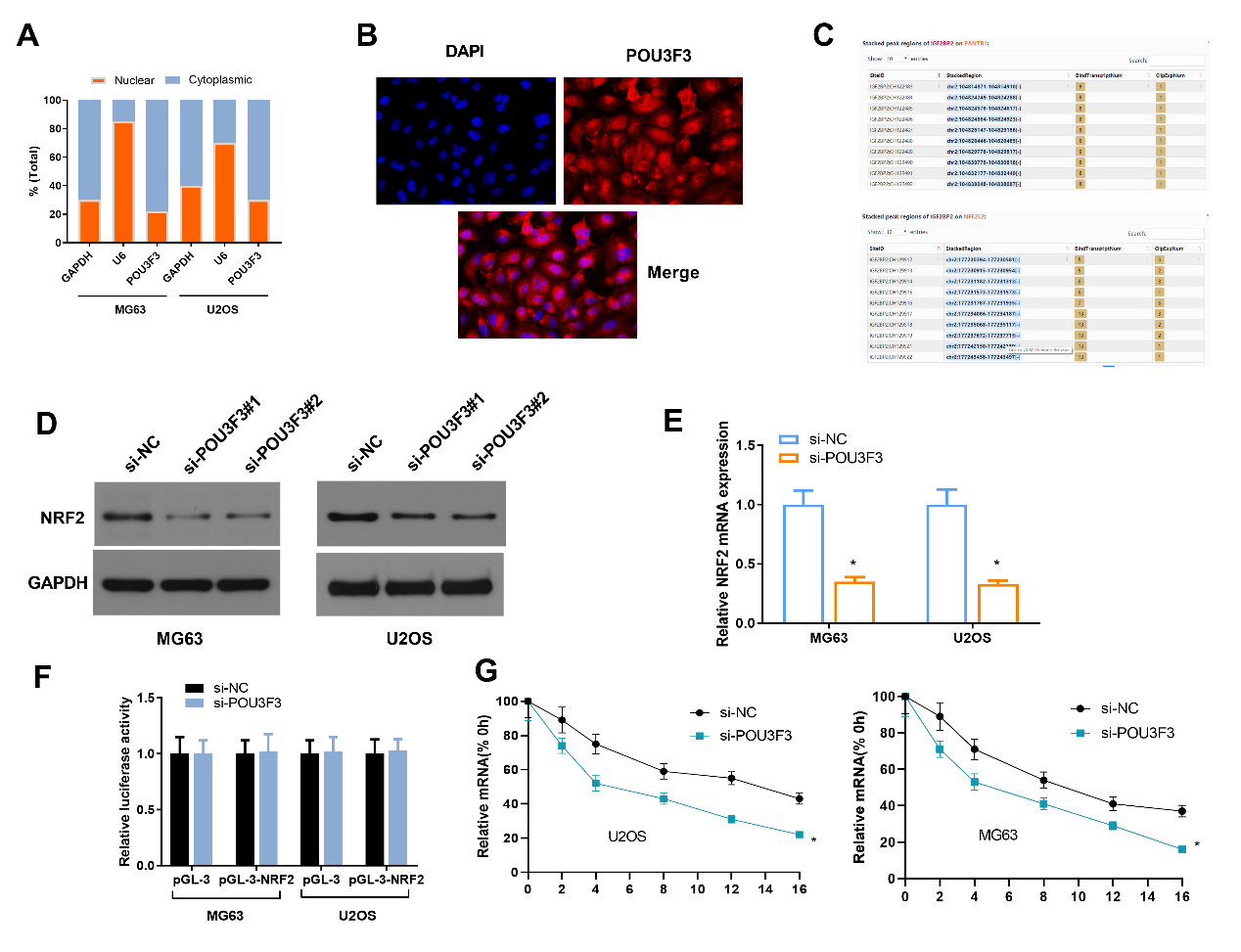


**Supplementary figure 4** POU3F3 exerted regulatory effects on the stability of NRF2 mRNA through post-transcriptional pathways. (A) Results of subcellular fractionation assay and RT-qPCR demonstrated cytoplasmic accumulation of POU3F3 in MG63 and U2OS cell lines. (B) Immunofluorescence results demonstrated that POU3F3 expressed primarily in the cytoplasm of osteosarcoma cells. (C) Interaction network illustrating the reciprocal association between POU3F3 and RNA-binding protein IGF2BP2, along with NRF2. (DE) Western blotting (D) and qRT-PCR (E) confirmed the influence of POU3F3 on NRF2 expression at both mRNA and protein levels. (F) Luciferase reporter assays investigating the effect of POU3F3 on NRF2 transcriptional activity, revealing no significant modulation. (G) Evaluation of NRF2 mRNA stability in response to POU3F3 knockdown, measured by qRT-PCR following actinomycin D treatment.


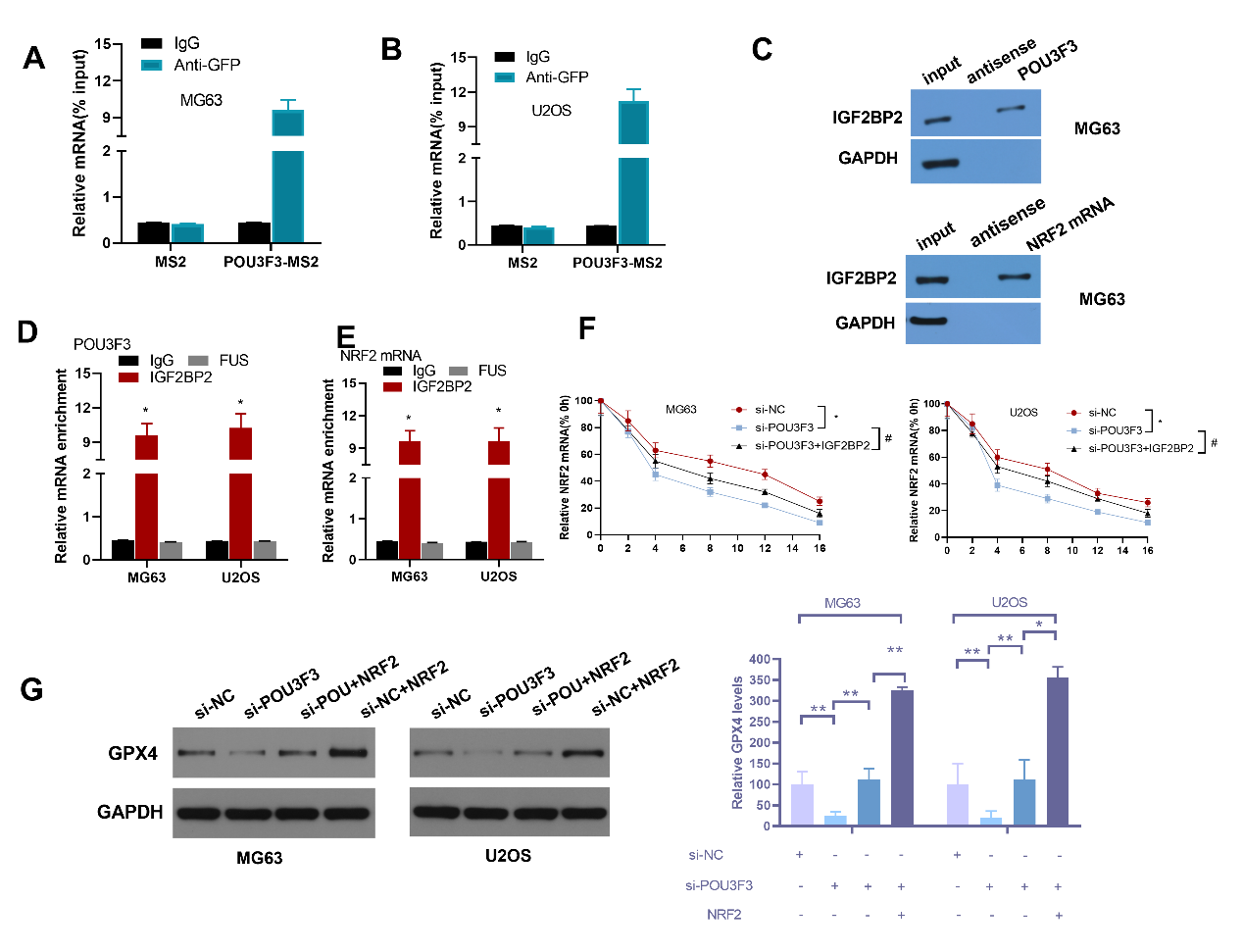


**Supplementary figure 5** POU3F3 enhanced the NRF2 level by directly interacting with IGF2BP2. (AB) MS2 RIP assays unveiled the binding of POU3F3 to NRF2 in MG63 (A) and U2OS (B) cells. (C) RNA pull-down assays demonstrated increased presence of IGF2BP2 in the POU3F3-precipitated complex, as well as direct interaction between IGF2BP2 and NRF2 mRNA. (DE) RIP assays using IGF2BP2 antibody confirmed the co-precipitation of POU3F3 and NRF2 mRNA in OS cells. (F) Rescue experiments revealed the interplay between POU3F3, IGF2BP2, and NRF2 mRNA stability, where IGF2BP2 overexpression countered POU3F3 knockdown effects. (G) Analysis of the regulatory influence of the POU3F3/NRF2/GPX4 axis on GPX4 protein expression levels.
